# Identification of hip fracture patients at risk for postoperative mobilisation complications via handgrip strength assessment

**DOI:** 10.1007/s00402-021-03756-9

**Published:** 2021-01-23

**Authors:** Johannes Gleich, Daniel Pfeufer, Alexander M. Keppler, Stefan Mehaffey, Julian Fürmetz, Wolfgang Böcker, Christian Kammerlander, Carl Neuerburg

**Affiliations:** Department of General, Trauma and Reconstructive Surgery, University Hospital, LMU Munich, Marchioninistr. 15, 81377 Munich, Germany

**Keywords:** Hip fracture, Gait analysis, Handgrip strength, Mobilisation, Outcome prediction

## Abstract

**Introduction:**

Treatment of older adult hip fracture patients can be challenging and requires early postoperative mobilisation to prevent complications. Simple clinical tools to predict mobilisation/weight-bearing difficulties after hip fracture surgery are scarcely available and analysis of handgrip strength could be a feasible approach. In the present study, we hypothesised that patients with reduced handgrip strength show incapability to follow postoperative weight-bearing instructions.

**Materials and methods:**

Eighty-four patients aged ≥ 65 years with a proximal femur fracture (trochanteric, *n* = 45 or femoral neck, *n* = 39), who were admitted to a certified orthogeriatric center, were consecutively enrolled in a prospective study design. Five days after surgery (intramedullary nailing or arthroplasty), a standardised assessment of handgrip strength and a gait analysis (via insole forcesensors) was performed.

**Results:**

Handgrip strength showed positive correlation with average peak force during gait on the affected limb (0.259), postoperative Parker Mobility Score (0.287) and Barthel Index (0.306). Only slight positive correlation was observed with gait speed (0.157). These results were congruent with multivariate regression analysis.

**Conclusion:**

Assessment of handgrip strength is a simple and reliable tool for early prediction of postoperative mobilisation complications like the inability to follow weight-bearing instructions in older hip fracture patients. Follow-up studies should evaluate if these findings also match with other fracture types and result in personalised adjustment of current aftercare patterns. In addition, efforts should be made to combine objectively collected data as handgrip strength or gait speed in a prediction model for long-term outcome of orthogeriatric patients.

## Introduction

Hip fractures are a life-changing event especially in older people and are associated with a high risk of mortality. According to latest epidemiologic data, related health loss expressed as disease adjusted life years (DALY) is expected to double from 2020 to 2040 [6]. At worst, an independent and mobile person becomes completely dependent on external help after suffering a hip fracture [28]. This is explained by the complexity of these patients: age-related physiological changes and various comorbidities like sarcopenia, visual impairment or cardiovascular diseases aggravate a return to the prefracture status of mobility and independency in activities of daily living (ADL).

Different forms of orthogeriatric treatment were implemented over the last years to address these special needs and to improve outcome of elderly trauma patients [21]. One main treatment goal of this interdisciplinary approach is early mobilisation with full weight-bearing after surgery, as immobilisation leads to complications like pneumonia, urinary tract infections or muscle atrophy associated with an increased mortality [1, 8, 18].

To assess individuals’ status of mobility and activities of daily living, various scores like Parker Mobility Score or Barthel Index are approved for a long time [16, 20]. Although these scores are frequently used, they only represent subjective tests and are dependent on the cooperation of the patient. Especially with regard to physical activity, various studies indicate that there are discrepancies between questionnaires and objective measurements of physical activity and patients significantly overestimated their daily activities [12, 26]. To address the needs of elderly hip fracture patients sufficiently, objective parameters for evaluation of individual mobility and mobilisation are essential. Real-time assessment of weight-bearing on the affected limb with insole force sensors has proven to be a feasible approach and provides objective data as well as a biofeedback. Braun et al. could demonstrate, using these sensors, that adherence to weight-bearing instructions in elderly trauma patients is low [2]. Although full weight-bearing is generally the aim after hip fracture surgery in these patients, some fracture patterns require modification of weight-bearing instructions. Thus, early identification of patients at risk to follow weight-bearing instructions would be desirable, as it might change the choice of treatment.

Besides gait speed, which has shown a significant correlation with the risk of mortality, handgrip strength appears as a promising value to predict outcome in elderly patients [17, 27, 29]. It is commonly used as a simple clinical parameter for physical function and muscle strength assessment [7]. Following the European Working Group on Sarcopenia in Older People 2 (EWGSOP2) also probable presence of sarcopenia can be defined herewith [4]. Furthermore, handgrip strength was proven as prognostic factor after hip fracture surgery [5, 23].

The aim of this study was to connect these parameters and to evaluate if patients with deficient handgrip strength also show deficits to follow postoperative weight-bearing instructions. We hypothesised that correlation between handgrip strength, objective (weight-bearing on affected limb, postoperative gait speed) and subjective parameters (mobility/ADL scores) helps to identify patients at risk for mobilisation complications after hip fracture surgery.

## Methods

### Study population

All patients aged 65 years and older, who were admitted to our certified orthogeriatric unit at a level-one trauma center with a proximal femur fracture (femoral neck or trochanteric region) between October 2017 and April 2018 were consecutively included in this prospective observational study after written informed consent. The study followed the Declaration of Helsinki and was registered at the German Clinical Trials Register (DRKS) under DRKS00012800 after approval of the local ethics review committee (Ref.-No.: 214-16). Instructions of the STROBE panel were followed for arrangement of this manuscript.

Exclusion criteria were fracture fixation of a femoral neck fracture, cognitive disorders (dementia, delirium, etc., defined by Mini-Mental State Examination < 26), language barrier, preexisting immobility and additional fractures of the upper or lower extremity.

Standardised and validated questionnaires were used to asses cognitive impairment (Mini-Mental State Examination), mobility and activities of daily living prior/after fracture [Parker Mobility Score (PMS)/Barthel Index (BI)]. Correct understanding and answering was verified by explicit inquiries for each patient.

Surgical treatment of trochanteric fractures was performed by intramedullary nailing [PFNA (Proximal Femoral Nail Antirotation); DePuy Synthes, Umkirch, Germany], femoral neck fractures were treated by arthroplasty (in case of total hip replacement: pinnacle acetabular cup, Biolox femoral head and Corail cemented stem/in case of bipolar hemiarthroplasty cemented Corail stem; DePuy Synthes, Umkirch, Germany).

On the first day after hip fracture surgery, all included patients were mobilised by physiotherapists with full weight-bearing as possible and training was continued each day. No other mobility restrictions were recommended, all patients received same postoperative care including standardised pain medication following WHO treatment guidelines, which was adjusted individually to facilitate painless walking.

### Assessment of handgrip strength and gait analysis

Standardised assessment was performed for handgrip strength and gait analysis on the fifth postoperative day. Handgrip strength was measured with a JAMAR Hydraulic Hand Dynamometer (Sammons Preston Inc., Bolinbrook, IL, USA) at the second handle position. Patients were in seating position, shoulder adducted and in neutral rotation, elbow flexed at 90 degrees, wrist between 0 and 30 degrees of flexion and between 0 and 15 degrees of ulnar deviation. Following instruction through the physiotherapy staff, three attempts of maximal voluntary squeezing were performed on both hands and highest result of dominant hand used for statistical analysis. Cut-off for probable presence of sarcopenia was set at 16 kg for woman/27 kg for men regarding to EWGSOP2 recommendation.

**Fig.1:**
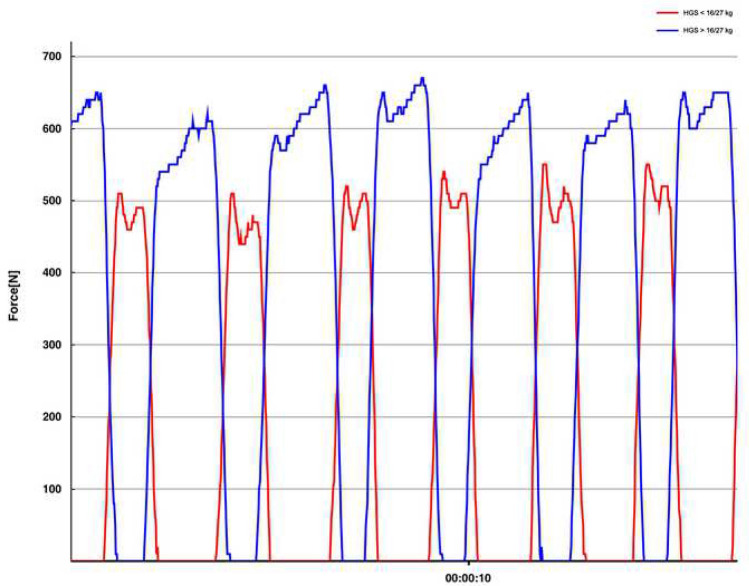
Exemplary illustration of load assessment on the affected leg for two male participants with handgrip strength above (blue)/below (red) EWGSOP2 cut-off (*x*-axis:time in seconds/*y*-axis: loading in N)

Insole force sensors (loadsoal^®^, Novel GmbH, Germany), fitted to the individuals foot size were placed in both shoes and used for gait analysis on a predefined walk of 40 m distance (starting from a chair, level walking, turning and returning to the chair). If necessary, the patient could use a walking aid of choice. Real-time data transmission (via Bluetooth to a tablet computer) of multiple parameters like gait speed, average peak force (average of maximum force values over the entire gait analysis in each step) and loading rate for each foot allows immediate analysis and storage (Fig. 1). Plantar force (in N) was measured in static and dynamic situations, the foot was scanned with up to 200 Hz and the capacitive sensors covered the complete plantar surface of the foot.

### Statistical analysis

Statistical analysis was performed using R (R Core Team 2019, R Foundation for Statistical Computing, Vienna, Austria). Normally distributed continuous variables are reported as mean with standard deviation (SD), categorical data as absolute frequency with a percentage distribution. Pearson and Spearman correlation coefficients (depending on data distribution) were used to analyse the association of handgrip strength with postoperative mobility (PMS, BI, average peak force loading on the affected leg, gait speed). Additionally, multivariate linear regression analysis, adjusted for age, gender and BMI, was performed to evaluate the influence of handgrip strength. Significance was set at a level of α = 0.05.

## Results

### Study population

In total, 122 patients were screened for eligibility, 84 of them met the inclusion criteria. Mean age was 79.71 (SD ± 6.769) years, 66.7% (*n* = 56) were female. Average ASA score was 2.67 (SD ± 0.545) and mean BMI 23.195 (SD ± 3.76). Most of the patients used a walking aid for postoperative mobilisation, only 4.8% were able to walk free. Barthel Index and Parker Mobility Score decreased significantly compared to prefracture/-operative status. A mean handgrip strength of 19.275 kg (SD ± 7,138) was observed, 59.5% (*n* = 50) of the patients were defined for probable presence of sarcopenia according to the EWGSOP2 criteria (see Table 1 for additional information).

**Table 1:** Patient characteristics (mean ± standard deviation (SD)/numbers with percentage in parentheses)

Characteristic	
Age (years)	79.71 ± 6.769
Female sex, *n*	56 (66.7%)
ASA score	2.67 ± 0.545
MMSE	29.17 ± 1.191
BMI (kg/m^2^)	23.195 ± 3.76
Type of fracture, *n*	
Trochanteric	45 (53.6%)
Femoral neck	39 (46.4%)
Type of treatment, *n*	
Intramedullary nailing	45 (53.6%)
Hemiarthroplasty	23 (27.4%)
Total hip replacement	16 (19.0%)
Barthel index (pre/post op)	95.24 ± 8.640/64.58 ± 15.174
PMS (pre/post op)	8.04 ± 1.773/3.75 ± 1.783
Mean HGS (kg)	19.275 ± 7.138
HGS < 16 kg/ < 27 kg (w/m), *n*	34/16 (59.5% in total)
Use of walking aid (postop), *n*	
None	4 (4.8%)
Cruches	22 (26.2%)
Walking frame	58 (69.0%)

Radiographic examination presented good implant positioning and fracture reduction in all patients, no wound healing disorder or other condition preventing mobilisation occurred during clinical course.

### Association of handgrip strength and postoperative mobilisation/mobility parameters

**Fig. 2:**
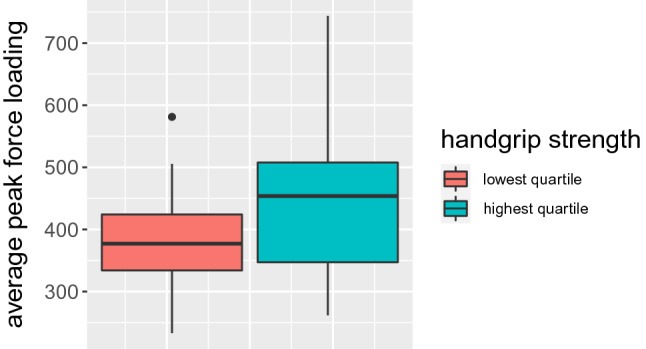
Boxplot presenting average peak force loading (N) for lowest/highest quartile of handgrip strength

Handgrip strength showed positive correlation with average peak force loading on the affected limb (Fig. 2), postoperative PMS and BI (see Table 2 for detailed information). Only slight positive correlation was observed between handgrip strength and postoperative gait speed. These findings were congruent with multivariate regression analysis (see Table 3 for detailed information).

**Table 2:** Handgrip strength correlation (r) with mobility parameters

Variable	Correlation (*r*)
Parker mobility score	0.287
Barthel index	0.306
Average peak force loading affected leg (N)	0.259
Gait speed	0.157

**Table 3:** Multivariate regression analysis of handgrip strength and mobility parameters

Variable	Coefficient (95% CI)	*p*
Parker mobility score	0.115 (0.046–0.185)	0.001
Barthel index	0.818 (0.208–1.427)	0.009
Average peak force loading affected leg (N)	6.855 (2.169–11.539)	0.005
Gait speed	0.004 (− 0.002–0.009)	0.222

## Discussion

Prompt mobilisation with adherence to weight-bearing instructions is essential to avoid in-hospital and long-term complications of older hip fracture patients. Early objective detection of patients at risk for mobilisation complications therefore is crucial, yet it can be challenging at the same time. Given the findings of the present study, assessment of handgrip strength could be used as simple and easy to implement identification tool to predict difficulties in mobilisation/weight-bearing in elderly hip fracture patients.

More than half of the study population presented with probable concomitant sarcopenia regarding to the EWGSOP2 criteria. This is higher than recent studies assessed in the perioperative setting, but consistent with results in postoperative setting [3, 24]. The observed high prevalence could be one major reason for recurrent falls following hip fracture surgery. Therefore, our findings should lead to a higher awareness for the presence of sarcopenia in acute care setting of orthogeriatric patients. Following the EWGSOP2 criteria, in clinical practice, detection of low muscle strength is enough to start further assessment and interventions (evaluation of nutritional status, administration of high caloric food, specific designed exercise programs [13, 25]). To determine muscle mass more precisely and confirm the diagnosis of sarcopenia, CT-based measurements could be used in addition, as a CT scan is performed in numerous patients at admission for supplementary fracture pattern evaluation and therefore is easily available [22].

Positive correlation of handgrip strength with subjective and objective parameters of postoperative mobility (PMS, loading on affected leg) and activities of daily living (BI) was observed in the present study. Correlation with peak force loading indicates that patients with superior handgrip strength can bear more weight on the affected limb during mobilisation and are able to follow the instruction of full weight-bearing. Vice versa, patients with reduced handgrip strength are more likely incapable to follow these instructions and therefore at risk for inappropriate surgical treatment and aftercare. Depending on the fracture pattern, modification of treatment concepts could be necessary. There is large consensus that aftercare treatment of hip fracture patients should follow full weight-bearing instructions. Fracture fixation of tibia plateau or pilon fractures frequently requires partial weight-bearing, whereas compliance to recommended weight-bearing limits in older trauma patients is low [2]. Taking findings of the present study into account, patients with reduced preoperative handgrip strength should be considered for joint replacement or joint fusion surgery in these fracture patterns to allow full weight-bearing. While previous studies evaluating the adherence of older trauma patients to weight-bearing recommendations with force sensors showed smaller number of patients, this study provides objective insights in a larger collective [2, 9].

Other than expected handgrip strength showed only slight positive correlation with postoperative gait speed. This is in contrast to recent findings by Orwig et al. [19]. One possible explanation might be found in different times of acquisition; in the present study, gait speed was assessed five days after surgery when patients still suffer from pain and toddle with following slow gait speed, whereas the acquisition period by Orwig et al. was two and six months after surgery. Although early identification of delayed mobilisation is essential, which favours analysis five days after surgery, selective correlation of grip strength with gait analysis/relevant scores at this time has to be regarded as a weakness of the present study. For future studies, a longitudinal observation, from admission to hospital until retrieval of individuals’ status of mobility, would be desirable to prove results and encourage long-term prediction of outcome via handgrip strength analysis.

Future combination of objective parameters like gait speed, weight-bearing on the affected limb and handgrip strength could lead to a prediction model for postoperative and long-term mobility. As recurrent falls are common following hip fracture, this model could also be used for secondary fracture prevention by identification of patients with low muscle strength and gait speed [15]. Lindemann et al. could show, that simple assessment of gait speed by visual categorisation is possible with reliable results, so even smaller hospitals could use the prediction model without technical expense [14]. In consequence, this should affect aftercare of each patient and lead to an individualised treatment plan. Given current technological developments, also individualised feedback regarding the recovery process could be possible beyond hospital stay with wearable devices and connected apps in the long run [10]. Regarding to Klenk et al. sensor-based assessment of physical activity is applicable for individual progress monitoring and might become an effective approach to further improve outcome in hip fracture patients [11].

To the best of the authors knowledge, the present trial remains the first study in which handgrip strength assessment in hip fracture patients was correlated with objective weight-bearing data, obtained with mobile sensors. Key findings are positive correlation of handgrip strength with postoperative weight-bearing and mobility measurements. As a consequence, handgrip strength assessment should be performed for every orthogeriatric patient already at admission to identify patients at risk for mobilisation complications. In the future, it could be a predictive model for postoperative and long-term mobilisation and mobility, consisting of easily accessible parameters like gait speed, weight-bearing and handgrip strength.
